# Antidiabetic and Immunomodulatory Properties of Peptide Fractions from Sacha Inchi Oil Press-Cake

**DOI:** 10.3390/foods14071231

**Published:** 2025-03-31

**Authors:** Erwin Torres-Sánchez, Cristina Martínez-Villaluenga, Samuel Paterson, Blanca Hernández-Ledesma, Luis-Felipe Gutiérrez

**Affiliations:** 1 Facultad de Ciencias Agrarias, Universidad Nacional de Colombia, Bogotá 111321, Colombia; egtorressa@unal.edu.co; 2 Instituto de Ciencia y Tecnología de Alimentos y Nutrición (ICTAN-CSIC), 28040 Madrid, Spain; c.m.villaluenga@csic.es; 3 Instituto de Investigación en Ciencias de la Alimentación (CIAL-UAM-CSIC), 28049 Madrid, Spain; samuel.paterson@csic.es; 4 Instituto de Ciencia y Tecnología de Alimentos (ICTA), Universidad Nacional de Colombia, Bogotá 111321, Colombia

**Keywords:** bioactive peptides, bioavailability, bioinformatics, molecular docking, *Plukenetia volubilis*

## Abstract

Sacha inchi (SI) oil press-cake (SIPC), a by-product of the sacha inchi oil extraction process, represents a novel protein source with potential bioactive applications in food. In this study, a sacha inchi protein concentrate (SPC) derived from SIPC was subjected to simulated gastrointestinal digestion (SGID) using the INFOGEST 2.0 protocol. The resulting digests were fractionated by ultrafiltration (<3, 3–10, and >10 kDa), and the bioactive properties of the peptide fractions were evaluated. In vitro α-amylase inhibition was assessed, along with immunomodulatory markers (NO, IL-6, and TNF-α), in an ex vivo RAW 264.7 cell model. Both gastric and intestinal digests exhibited significant α-amylase inhibition (20–45%), with the <3 kDa intestinal fraction showing the highest inhibition (45% at 20 mg/mL). Both gastric and intestinal <3 kDa fractions reduced NO production in RAW 264.7 macrophages subjected to a lipopolysaccharide challenge. HPLC-MS/MS analysis facilitated de novo sequencing of the peptide fractions, identifying 416 peptides resistant to SGID through the find-pep-seq script, which were further assessed in silico for toxicity, allergenicity, and bioavailability, revealing no significant risks and potential drug-likeness development. Molecular docking simulations of three peptides (RHWLPR, RATVSLPR, and QLSNLEQSLSDAEQR) with α-amylase and four peptides (PSPSLVWR, RHWLPR, YNLPMLR, and SDTLFFAR) with the TLR4/MD-2 complex suggesting potential roles in α-amylase inhibition and anti-inflammatory activity, respectively. The findings suggest that SI protein concentrates could be used in functional foods to prevent starch breakdown through α-amylase-inhibiting peptides released during digestion, reduce blood glucose, and mitigate inflammation and oxidative tissue damage.

## 1. Introduction

Sacha inchi (SI) (*Plukenetia volubilis*), an oilseed climbing plant from the Euphorbiaceae family, is native to the Amazon region and has gained global economic significance, particularly in Africa and Asia, due to the high quality of its oil, rich in essential fatty acids [1]. The by-product of SI oil extraction, known as SI oil press-cake (SIPC), contains 53–59% crude protein, and it is rich in AA such as lysine, histidine, and leucine. Despite lower levels of threonine and methionine, its AA composition meets FAO/WHO/UN nutritional recommendations, except for leucine and lysine [2]. SIPC is used in various food products and as protein isolates or hydrolysates [3,4]. Recently, bioactive peptides with antidiabetic and immunomodulatory properties derived from plant by-products have attracted attention due to their positive effects on non-communicable diseases like diabetes, cancer, and cardiovascular diseases [5,6,7]. Diabetes mellitus, particularly DM2, is a chronic metabolic disorder marked by hyperglycemia, and it is among the leading causes of death globally. DM2, which constitutes 85–95% of all diabetes cases, results from insulin resistance and impaired insulin secretion, leading to elevated blood glucose levels [8,9]. Traditional treatments, such as insulin injections, have limitations and side effects [10,11]. Consequently, there is a growing need for natural and food components with antidiabetic properties.

Inhibition of α-amylase plays a crucial role in regulating carbohydrate digestion and glucose absorption by limiting starch hydrolysis and reducing the availability of glucose substrates [8]. Previous studies have shown that protein hydrolysates and bioactive peptides from legumes effectively inhibit starch digestion and glucose absorption. For example, peptides from germinated soy protein subjected to SGID demonstrated dose-dependent inhibition of α-amylase, α-glucosidase, and DPP-IV enzymes with IC_50_ values ranging from 1.70–10.00 mg/mL [12]. Similarly, a protein hydrolysate from black beans exhibited high inhibitory potential against these enzymes [13]. In a pioneering study, SIPC-derived protein hydrolysates inhibited α-amylase by 10–55% at concentrations of 0.13–2.00 mg/mL [14], while hydrolysates produced with Protamex and alkaline proteases showed potent DPP-IV inhibitory peptides with IC_50_ values of 23.43–128.40 µmol/L [15]. These findings suggest that SIPC hydrolysates can be used in food to regulate postprandial blood glucose levels.

Additionally, SIPC protein hydrolysates exhibit high antioxidant activity, demonstrated by methods such as Fe^2+^ chelation, DPPH radical scavenging, and ferric-reducing antioxidant power [16]. Other studies have shown that SIPC-derived peptides have hypoglycemic and antioxidant properties through in vitro and ex vivo assays [17,18,19]. Furthermore, bioactive peptides from SIPC have shown immunomodulatory effects, enhancing macrophage activity and promoting NO and TNF-α production, which are key in immune responses [20].

However, the bioactivity of peptides as functional ingredients relies on their resistance to digestion, as structural changes may compromise their functionality. To date, no studies have reported using SGID to release peptide fractions from SI proteins with antidiabetic and immunomodulatory effects. Thus, this study aims to evaluate the in vitro α-amylase inhibition properties and ex vivo immunomodulatory markers (NO, IL-6, and TNF-α) in RAW 264.7 cells of peptide fractions obtained via the INFOGEST 2.0 protocol. Additionally, in silico analyses were performed to assess the toxicity, allergenicity, bioavailability, and molecular docking of selected peptides with receptors from the RCSB Protein Data Bank.

## 2. Materials and Methods

### 2.1. Materials

Defatted SIPC was obtained from SumaSach’a (Mosquera, Cundinamarca, Colombia). The ultrafiltration membrane system was supplied by Sartorius^®^ (Vivaflow^®^ TFF Cassette, Göttingen, Germany). The RAW 264.7 macrophage cell line was sourced from the American Type Culture Collection (Rockville, MD, USA). DMEM, FBS, penicillin, and streptomycin were provided by Biowest (Kansas City, MO, USA). IL-6 and TNF-α ELISA kits were obtained from Diaclone SAS (Besançon, France). The 96-well and 48-well plates were supplied by Sarstedt AG & Co. (Nümbrecht, Germany) and Corning Costar Corporation (Corning, NY, USA), respectively. Water used in the experiments was obtained through a Milli-Q water purification system (Millipore, Bedford, MA, USA). Other reagents and solvents were of analytical grade and supplied by Sigma-Aldrich (St. Louis, MO, USA) and Merck (Kenilworth, NJ, USA).

### 2.2. SGID and Peptide Fractionation

The in vitro INFOGEST 2.0 harmonized protocol [21] was used, following the methodology described [19]. Saliva was collected from 20 healthy volunteers. To achieve SGID, 3 g of an SPC (*n* = 6)—obtained by mixing SIPC with deionized water (1:10 *w*/*v*) under alkaline conditions (pH 11.0, adjusted with NaOH), stirring for 1 h at 800 rpm and 60–70 °C, centrifuging, then neutralizing, diafiltrating, and freeze-drying the supernatant (30 °C, 0.08–0.1 mbar, 24 h) [19]—was dissolved in human saliva (1:5, *v*/*v*) and subjected to the oral phase for 5 min. The mixture was then diluted with simulated gastric fluid (1:10, *v*/*v*) and subjected to the gastric phase with pepsin (EC 3.4.23.1) at an enzyme-to-substrate ratio (E:S) of 1:60 (*w*/*w*) for 2 h. Samples (gastric digest GD, *n* = 3) were collected after pepsin inactivation (pH adjustment to 7.0 with NaOH or HCl, heating at 95 °C for 15 min) and freezing at −40 °C. After completing the gastric phase, the remaining samples were mixed with simulated intestinal fluid, pancreatin at an E:S ratio of 1:1.2 (*w*/*w*), and bile salts at a ratio 1:30 (*w*/*w*) for 2 h. Samples (intestinal digest, ID, *n* = 3) were collected after pancreatin inactivation (heating at 95 °C for 15 min) following the same freezing protocol.

Subsequently, samples were processed using the Sartorius^®^ ultrafiltration system with 3 kDa and 10 kDa membranes to produce GD3/ID3 (<3 kDa), GD2/ID2 (3–10 kDa), and GD1/ID1 (>10 kDa) fractions, following the methodology reported in the literature [22]. Digestion blanks were also fractionated. All samples were freeze-dried and stored at −18 °C.

### 2.3. In Vitro α-Amylase Inhibition Assay

Samples were dissolved in 20 mM phosphate buffer containing 6.7 mM NaCl (pH 6.9) to achieve final concentrations ranging between 1 and 20 mg/mL. Subsequently, 50 µL of sample (1–20 mg/mL), positive control (2 mM acarbose), or negative control (phosphate buffer) was mixed with 100 µL of α-amylase-A3176-1MU^®^ solution (2 U/mL in 25 mM Tris-HCl buffer, pH 7.5, containing 100 mM KCl). The mixture was incubated at 20 °C for 5 min at 1000 rpm in a Thermomixer^TM^ orbital shaker (Eppendorf Ibérica, Madrid, Spain). Following incubation, 100 µL of 1% soluble starch was added and incubated for 6 min. Afterward, 100 µL of dinitrosalicylic reagent (96 mM with 2 M sodium potassium tartrate and 2 M NaOH) was added, and the mixture was incubated 15 min at 100 °C. The final volume was adjusted to 1000 µL with distilled water, and 200 µL of the reaction mixture was transferred to 96-well plates for absorbance measurement at 540 nm using a Synergy^TM^ HT reader (Biotek, Winooski, VT, USA). A standard curve was used to determine maltose concentration (mg equivalents), and the percentage of α-amylase inhibition was calculated using Equation (1). Phosphate buffer was used as blank.

(1) $$\alpha -\mathrm{amylase}\;\mathrm{inhibition}\;(\%)=100-(\frac{Maltose\;\left(mg\right)\;of\;sample-Maltose\;\left(mg\right)\;of\;blank\;sample\times 100}{Maltose\;\left(mg\right)\;of\;negative\;control}),$$

### 2.4. Modulatory Effects in RAW 264.7 Cell Model

#### 2.4.1. Culture

RAW 264.7 murine macrophages were routinely cultured in T75 flasks using DMEM supplemented with 10% FBS and 1% penicillin/streptomycin/amphotericin. The cells were maintained at 37 °C in a humidified environment with 5% CO_2_ and 95% air. Once the cells reached 80–90% confluence, they were harvested by gentle scraping, washed with PBS, and centrifuged at 1000 rpm for 5 min to collect cell pellets. The pellets were then resuspended in DMEM. Cell counting was performed using an EVE™ Plus cell counter (NanoEntek, Seoul, Republic of Korea) to adjust the cell suspension density for subsequent assays.

#### 2.4.2. Determination of NO Production

To assess the impact of samples on NO release, the Griess assay was utilized with slight modifications based on established protocols [23]. RAW 264.7 cells were plated at a density of 5 × 10^4^ cells/well in 96-well plates and incubated at 37 °C for 24 h. Then, the medium was discarded, and the cells were treated with samples dissolved in FBS-free DMEM for 24 h at 37 °C. Negative controls consisted of cells treated with FBS-free DMEM, while positive controls received FBS-free DMEM supplemented with LPS at a concentration of 10 µg/mL. A volume of 100 µL of the supernatant was collected and combined with 100 µL of Griess reagent (containing 0.1% N-(1-napthyl)ethylenediamine dihydrochloride, 1% sulfanilamide, and 2.5% phosphoric acid) and incubated at room temperature for 15 min. Absorbance was measured at 540 nm using the Biotek Synergy™ HT (Biotek) plate reader. The concentration of NO was determined using a NaNO_2_ standard curve (3.125–100 µM).

#### 2.4.3. Cytokine Production

To evaluate the effect of the samples on the immune response of RAW 264.7 cells, cytokine release was measured. RAW 264.7 cells were plated at a density of 5 × 10^5^ cells/well in 48-well plates and incubated at 37 °C for 24 h. Then, the medium was removed, and the cells were treated with the samples dissolved in FBS-free DMEM for 24 h at 37 °C. Negative and positive controls were cells treated with FBS-free DMEM and FBS-free DMEM plus LPS (10 µg/mL), respectively. The supernatants were collected, and commercial ELISA kits were used to quantify IL-6 and TNF-α levels, following the manufacturer’s instructions. Absorbances were read at 450 nm using the Biotek Synergy™ HT (Biotek) plate reader. The concentration of cytokines was calculated using standard curves.

### 2.5. Peptide Sequencing and SGID-Resistant Peptides Identification

HPLC-MS/MS assays were conducted at the Proteomics Facility of the Centro de Biología Molecular Severo Ochoa (CBM-CSIC, Madrid, Spain). The analyses were conducted using an ion trap LTQ-Orbitrap-Velos-Pro hybrid mass spectrometer equipped with a nano-spray source, coupled to an Easy-nLC 1200 system (Thermo Scientific, Waltham, MA, USA), following the reported methodology [24]. Peptide identification was carried out by analyzing mass spectrometric data with the de novo PEAKS Studio V11.5 search engine (Bioinformatics Solutions Inc., Waterloo, ON, Canada). Peptides with an ALC score ≥ 85% were selected for further analysis.

To identify peptide sequences resistant to in vitro SGID, de novo peptide sequences from ID and those in the initial SPC were compared using the find-pep-seq script [15], calculating the cosine similarity measure (threshold of 0.95), and discarding paired peptide sequences with a length difference of more than 8 AA, following the reported methodology [19]. The SGID-resistant peptides identified were deduplicated and formatted into a FASTA file for subsequent bioinformatics analysis.

### 2.6. In Silico Analysis of SGID-Resistant Peptides

#### 2.6.1. Identification of Inhibitory α-Amylase Peptides

The position of certain AA at the C- or N-terminal is a crucial feature for α-amylase inhibitory peptides. SGID-resistant peptides (Section 2.5) were classified by length into short (2–5 AA), medium (6–10 AA), and long (>10 AA) categories. Additionally, medium and long peptides were classified based on the type of AA and its position at key terminal locations: ultimate (C1 and N1), penultimate (C2 and N2), and antepenultimate (C3 and N3) positions, according to the literature [25], selecting potential α-amylase inhibitors and analyzing them using the following in silico tools: PepCalc [26] to estimate the MW, isoelectric point (pI), net charge, and solubility; ADMETlab 2.0 [27] to predict the AMES and rat oral acute toxicity indices; and AllerCatPro 2.0 [28] to assess their potential allergenicity.

#### 2.6.2. Bioactivity and Bioavailability of Anti-Inflammatory Peptides

The peptides identified as resistant (Section 2.5) were screened to identify those most likely to be bioactive and bioavailable. The tools used included Peptide Ranker [29] to select peptides with probabilities greater than 0.75. The selected peptides were then analyzed using PreAIP [30] to predict their likelihood of being anti-inflammatory. ADMETlab 2.0 [27] was employed to assess the bioavailability of the peptides based on the Pfizer Rule for drug-likeness, as well as parameters for absorption, such as Caco-2 cell permeability and human intestinal absorption. Additionally, distribution parameters, including plasma protein-binding scores, and toxicity assessments based on rat oral acute toxicity and skin sensitization tests, were evaluated. AllerCatPro 2.0 [28] was used to assess the potential allergenicity of the peptides. Finally, the probability of the peptides being cell-penetrating peptides (CPP) was predicted using the MLCPP 2.0 software [31].

### 2.7. Molecular Docking with Resistant and Bioactive Peptides

#### 2.7.1. Resistant Peptides and α-Amylase

Ten peptides (five medium-sized and five long) were selected based on their structural characteristics, including AA type and position (Section 2.6.1), and subjected to molecular docking using a well-established methodology [32]. Autodock Vina (V1.1.2) [33] was employed to predict interactions between the peptides and α-amylase. The X-ray crystallographic structure of α-amylase (PDB ID: 5U3A) was obtained from the RCSB Protein Data Bank (https://www.rcsb.org/, accessed 27 July 2024) in PDB format. The peptide structures were drawn using Marvin JS 24.1.0 (Chemaxon Ltd., Budapest, Hungary) and saved in .pdb format with Avogadro V1.2.0 [34]. AutoDock Tools V1.5.7 (The Scripps Research Institute, Molecular Graphics Laboratory, La Jolla, CA, USA) was used to prepare the α-amylase structure by removing water molecules, adding polar hydrogens, and applying Kollman charges. Peptides were prepared by adding Gasteiger charges. The docking parameters were central coordinates (x = 8.561, y = 80.915, z = 149.924) and docking box size (x = 25, y = 25, z = 25).

#### 2.7.2. Resistant Peptides and TLR4/MD-2 Complex

The selected resistant peptides (Section 2.6.2) were subjected to molecular docking to predict interactions with the TLR4/MD-2 complex, following the methodology described in the literature [32,35]. The peptide structures were drawn as described in Section 2.7.1. The X-ray crystallographic structure of TLR4/MD-2 (PDB ID: 5IJD) was retrieved from the RCSB Protein Data Bank (accessed August 2024) in PDB format. Using UCSF Chimera, homodimer chains B/D of the protein were selected, and non-protein molecules were removed. AutoDock Tools V1.5.7 was used to prepare the TLR4/MD-2 complex and peptides, as described in Section 2.7.1. The docking parameters were central coordinates (x = 12.671, y = −14.606, z = 29.023) and docking box size (x = 44, y = 46, z = 56).

For both α-amylase and TLR4/MD-2 complex docking, the following parameters were applied: energy range = 3, exhaustiveness = 8, and number of modes = 9. Each ligand was docked in 10 rounds, and the conformation with the lowest binding energy was selected for further analysis. Structural visualizations were generated using Discovery Studio Visualizer V24.1.0.23298 (Dassault Systèmes Biovia Corp., San Diego, CA, USA) [36].

### 2.8. Statistical Analysis

The in vitro and ex vivo experiments were carried out under completely randomized designs, with each parameter evaluated independently and measurements performed in triplicate at minimum. ANOVA and test of significance (Duncan’s multiple range test) were performed using SAS^®^ OnDemand Software (V3.81) (Cary, NC, USA: SAS Institute Inc., accessed in July 2024). The homogeneity of variances was assessed using Levene’s test. The normality of the residuals was evaluated using the Shapiro–Wilk test. Differences were considered statistically different at *p* < 0.05.

## 3. Results and Discussion

### 3.1. α-Amylase Inhibition Activity

**Figure 1:**
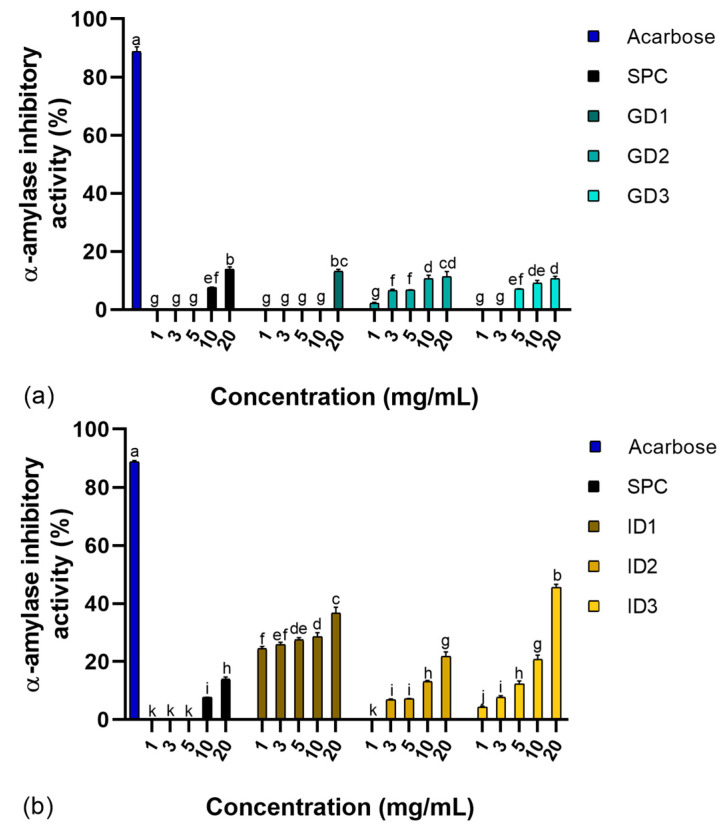
α-Amylase inhibitory activity of a sacha inchi protein concentrate (SPC) and their fractioned digests. (**a**) Gastric digests (GD). (**b**) Intestinal digests (ID). ^a–k^ Different letters indicate significant differences (Duncan’s multiple range test, *p* < 0.05). SPC: Sacha inchi protein concentrate; GD1 and ID1: Digest fractions (>10 kDa); GD2 and ID2: Digest fractions (3–10 kDa); GD3 and ID3: Digest fractions (<3 kDa). Acarbose was used at 2 mM.

SPC and their GD and ID digests, fractionated by molecular size, were evaluated for their capacity to inhibit α-amylase activity using a standard enzymatic in vitro assay. The results are shown in Figure 1.

Synthetic acarbose (positive control) demonstrated significantly higher inhibitory activity (~87%, *p* < 0.05) compared to the other samples. SPC, along with fractions GD1, GD2, and GD3, exhibited approximately 13% inhibition at 20 mg/mL. Both GD2 and GD3 fractions displayed dose-dependent inhibitory activity, with GD2 effective at concentrations of 3–20 mg/mL and GD3 at 5–20 mg/mL (Figure 1a). The observed α-amylase inhibitory activity aligned with previously reported values for SI peptides, which showed inhibition rates of 14% and 32% at concentrations of 2 and 5 mg/mL, respectively, following pepsin digestion [18]. However, our results were lower than those reported for SI protein concentrate hydrolyzed with pepsin and fractionated to obtain 1–3 kDa peptides, which achieved ~55% inhibition at 4 mg/mL [14]. This difference in inhibitory activity may stem from variations in peptide size and composition due to differing experimental conditions. For example, the use of more refined molecular weight fractions (e.g., 1–3 kDa) in the cited study likely improved inhibitory potential.

Following gastrointestinal digestion, the samples demonstrated significantly stronger α-amylase inhibition (*p* < 0.05), with a clear dose-dependent response (Figure 1b). The ID3 fraction exhibited the highest inhibitory activity, achieving 45.6% inhibition at 20 mg/mL. In contrast, the ID1 fractions showed significantly higher inhibition (*p* < 0.05) across all tested concentrations (1–20 mg/mL), ranging from 24.5% to 36.7%. No significant differences (*p* > 0.05) were observed between the ID2 and ID3 fractions at 10 and 20 mg/mL, both exhibiting approximately 21% inhibition. These results indicate that increased hydrolysis during gastrointestinal digestion, coupled with molecular fractionation, enhanced α-amylase inhibitory activity. These findings align with previous studies on SGID of quinoa (*Chenopodium quinoa*), where α-amylase inhibition increased during the duodenal phase, reaching 50% inhibition at 5 mg/mL with an intestinal digest of molecular weight <5 kDa derived from a protein concentrate [37]. The stronger α-amylase inhibitory activity of the ID3 fraction (45.6% inhibition at 20 mg/mL) may be attributed to its optimal peptide size, charge, and hydrophobicity. Peptides within the 3–10 kDa range likely balance binding affinity and accessibility to α-amylase’s active site. Their charge distribution may enhance electrostatic interactions, while greater hydrophobicity could improve binding to the enzyme’s hydrophobic regions. Additionally, these peptides may exhibit resistance to digestion, enabling them to remain functionally active throughout the gastrointestinal process [8].

### 3.2. NO Production

The Griess assay was used to assess the effects of samples on NO production in RAW 264.7 cells. Under basal conditions, treatment with SPC (63 μg/mL) (data not plotted) did not significantly alter NO production compared to the control (1.21 ± 0.06 vs. 1.16 ± 0.01 μM; *p* > 0.05). However, SPC at 125 μg/mL increased NO levels to 1.59 ± 0.09 μM, exceeding the control but remaining lower than LPS at 10 μg/mL (10.83 ± 0.62 μM; *p* < 0.05). Under stimulated conditions, SPC at 63 μg/mL produced NO levels (10.50 ± 0.25 μM) comparable to LPS (*p* > 0.05), while SPC at 125 μg/mL plus LPS resulted in lower NO levels (9.05 ± 0.58 μM; *p* < 0.05).

**Figure 2:**
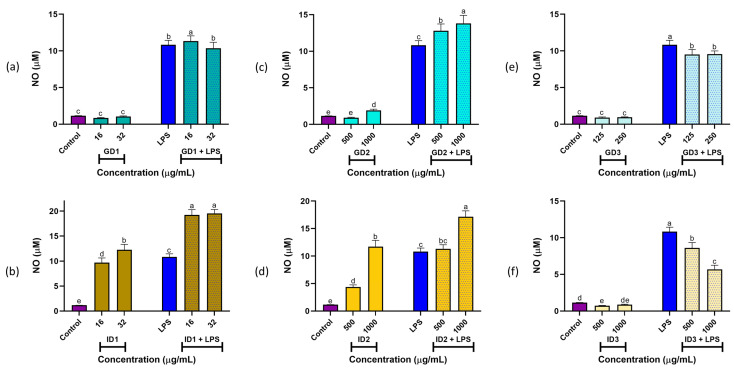
Nitric oxide (NO) levels (expressed as µM) in RAW264.7 macrophages under basal and stimulated conditions. (**a**,**c**,**e**) show gastric digest fractions GD1, GD2, and GD3, respectively. (**b**,**d**,**f**) show intestinal digest fractions ID1, ID2, and ID3, respectively. ^a–e^ Different letters indicate significant differences (Duncan’s multiple range test, *p* < 0.05). Lipopolysaccharide (LPS) stimulus was 10 μg/well. SPC: Sacha inchi protein concentrate; GD1 and ID1: Gastric and intestinal digest fractions (>10 kDa), respectively; GD2 and ID2: Gastric and intestinal digest fractions (3–10 kDa), respectively; GD3 and ID3: Gastric and intestinal digest fractions (<3 kDa), respectively.

The main results for NO production from gastric and intestinal digests of SPC are shown in Figure 2. For gastric digests under basal conditions, treatments with GD1 (16 and 32 μg/mL; Figure 2a), GD2 (500 and 1000 μg/mL; Figure 2c), and GD3 (125 and 250 μg/mL; Figure 2e) had no significant effect on NO production compared to the control (*p* > 0.05), except for GD2 at 1000 μg/mL, which was still significantly lower than LPS (*p* < 0.05). Under stimulated conditions, GD1 (16 μg/mL) produced significantly higher NO levels (*p* < 0.05) compared to LPS and GD1 (32 μg/mL), which were similar (*p* > 0.05). GD2 (500 and 1000 μg/mL) resulted in the highest NO levels, exceeding all other treatments. In contrast, GD3 (125 and 250 μg/mL) significantly reduced LPS-induced NO production in macrophages (*p* < 0.05).

For intestinal digests under basal conditions, treatments with ID1 (16 and 32 μg/mL; Figure 2b) and ID2 (500 and 1000 μg/mL; Figure 2d) significantly increased NO levels compared to the control (*p* < 0.05). Under stimulated conditions, NO production for both ID1 and ID2 was significantly higher than LPS (*p* < 0.05). However, ID3 (500 and 1000 μg/mL; Figure 2f) significantly inhibited NO production compared to both the control and LPS (*p* < 0.05).

NO production is a key biomarker of inflammation in macrophages’ cytotoxic responses, enhancing their defensive capabilities [23]. Notably, the GD3 and ID3 digests did not activate immune responses in macrophages under basal conditions (no significant NO production). However, GD3 and ID3 peptide fractions may modulate anti-inflammatory responses by reducing NO production in the presence of pro-inflammatory stimuli like LPS. These findings align with previous studies showing that albumin protein fractions from SI inhibit LPS-induced macrophage activation [20]. Complete SGID and ultrafiltration promote extensive hydrolysis, generating small molecules with anti-inflammatory effects. This was demonstrated when LPS-stimulated RAW 264.7 cells were treated with extruded amaranth hydrolysates (0.25–1.00 mg/mL) produced using pepsin/pancreatin, which inhibited NO production by 11.6% to 60% [38]. These results highlight the potential of SI digests to prevent chronic inflammation and reduce oxidative damage to cells and tissues, offering significant benefits in inflammatory or infectious conditions.

### 3.3. Cytokines IL-6 and TNF-α Production

To evaluate IL-6 and TNF-α production in macrophages treated with SPC and its digest fractions, ELISA assays were conducted. Under basal conditions, SPC (63 and 125 μg/mL) did not significantly alter (*p* > 0.05) IL-6 production compared to the control (0.43 ± 0.06 ng/mL). However, under stimulated conditions, SPC (63 and 125 μg/mL) increased IL-6 levels to 20.97 ± 0.3 ng/mL (*p* < 0.05), comparable to LPS (*p* > 0.05). In both basal and stimulated conditions, TNF-α production by RAW 264.7 cells was significantly higher than the control (*p* < 0.05) and similar to LPS (*p* > 0.05) when treated with SPC (63 and 125 μg/mL).

**Figure 3:**
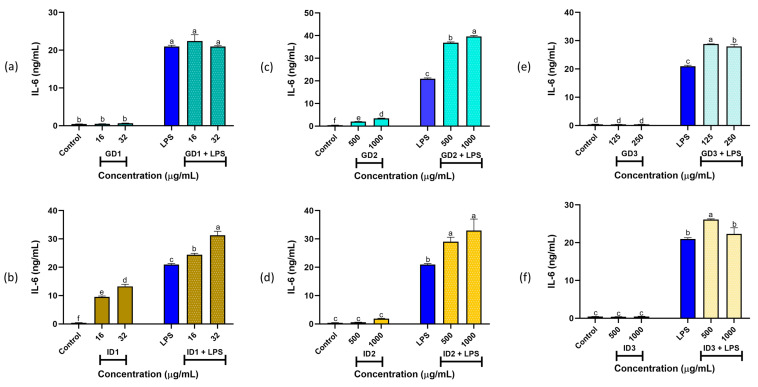
Murine IL-6 (interleukin 6) levels (expressed as ng/mL) in RAW264.7 macrophages under basal and stimulated conditions. (**a**,**c**,**e**) show gastric digest fractions GD1, GD2, and GD3, respectively. (**b**,**d**,**f**) show intestinal digest fractions ID1, ID2, and ID3, respectively. ^a–f^ Different letters indicate significant differences (Duncan’s multiple range test, *p* < 0.05). Lipopolysaccharide (LPS) stimulus was 10 μg/well. SPC: Sacha inchi protein concentrate; GD1 and ID1: Gastric and intestinal digest fractions (>10 kDa), respectively; GD2 and ID2: Gastric and intestinal digest fractions (3–10 kDa), respectively; GD3 and ID3: Gastric and intestinal digest fractions (<3 kDa), respectively.

**Figure 4:**
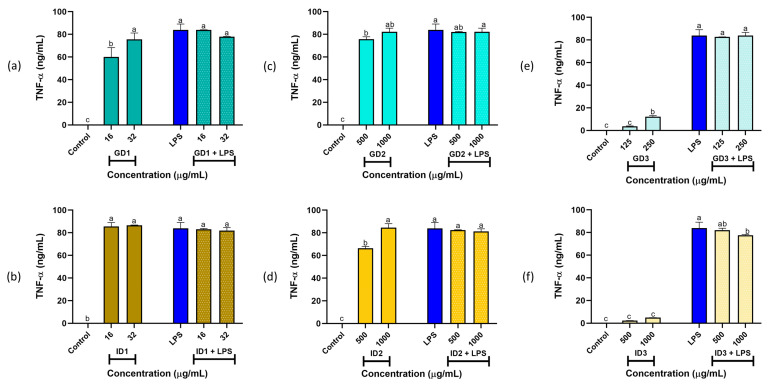
Murine TNF-α (tumor necrosis factor alpha) levels (expressed as ng/mL) in RAW264.7 macrophages under basal and stimulated conditions. (**a**,**c**,**e**) show gastric digest fractions GD1, GD2, and GD3, respectively. (**b**,**d**,**f**) show intestinal digest fractions ID1, ID2, and ID3, respectively. ^a–c^ Different letters indicate significant differences (Duncan’s multiple range test, *p* < 0.05). Lipopolysaccharide (LPS) stimulus was 10 μg/well. Doses below the dashed green line are toxic. SPC: Sacha inchi protein concentrate; GD1 and ID1: Gastric and intestinal digest fractions (>10 kDa), respectively; GD2 and ID2: Gastric and intestinal digest fractions (3–10 kDa), respectively; GD3 and ID3: Gastric and intestinal digest fractions (<3 kDa), respectively.

For the digest fractions, IL-6 and TNF-α production in RAW 264.7 cells are shown in Figure 3 and Figure 4, respectively. Under basal conditions, treatment with GD1 (16 and 32 µg/mL; Figure 3a), GD3 (125 and 250 µg/mL; Figure 3e), ID2 (500 and 1000 µg/mL; Figure 3d), and ID3 (500 and 1000 µg/mL; Figure 3f) resulted in IL-6 levels similar to the control (*p* > 0.05). In contrast, GD2 (500 and 1000 µg/mL; Figure 3c) and ID1 (16 and 32 µg/mL; Figure 3b) induced higher IL-6 production than the control but lower than LPS (*p* > 0.05). Under stimulated conditions, all fractions (GD1, GD2, GD3, ID1, ID2, and ID3) at both tested concentrations produced IL-6 levels equal to or greater than those induced by LPS (*p* < 0.05).

Macrophages are key components of the innate immune system, serving as the first line of defense against pathogen invasion. Upon activation, macrophages release pro-inflammatory cytokines such as IL-6 and TNF-α to enhance their defensive capabilities [39]. Notably, the fractions GD1, GD3, ID2, and ID3 exhibit anti-inflammatory properties, as they do not stimulate IL-6 production by macrophages under basal conditions. However, under stimulated conditions, these gastric and intestinal fractions fail to counteract the effects of LPS; instead, they promote the initiation of the macrophage immune response by increasing IL-6 production in the presence of an infectious agent.

On the other hand, as shown in Figure 4, TNF-α production for all samples, under both basal and stimulated conditions, was greater than the control and comparable to that induced by LPS (*p* < 0.05), except for GD3 and ID3. Notably, under basal conditions, treatment with GD3 (125 μg/mL; Figure 4e) and ID3 (500 and 1000 μg/mL; Figure 4f) resulted in TNF-α levels similar to the control (*p* > 0.05). Importantly, the ID3 (1000 μg/mL) fraction significantly reduced TNF-α production (*p* < 0.05) in the presence of LPS. These findings highlight the anti-inflammatory potential of the GD3 and ID3 fractions.

These results align with previous studies in which lunasin isolated and purified from quinoa (*Chenopodium quinoa*) significantly inhibited NO, TNF-α, and IL-6 production in LPS-stimulated RAW264.7 macrophages, reducing levels by up to 44.77%, 39.81%, and 33.50%, respectively, at a concentration of 0.40 g/L [40]. Similarly, a 5 kDa lunasin peptide (at 10 mM) derived from defatted soybean flour strongly inhibited pro-inflammatory markers in LPS-stimulated RAW264.7 macrophages, including NO and IL-6 production by 43% and 75.6%, respectively [41]. These findings demonstrate that low molecular weight peptide fractions exhibit potent anti-inflammatory properties.

### 3.4. De Novo Peptides Sequencing and SGID-Resistant Peptides Identification

**Figure 5:**
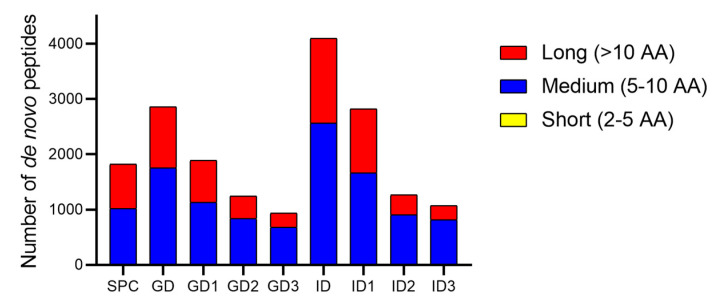
Number of de novo peptides of a sacha inchi protein concentrate (SPC) and their digests classified by the number of amino acid (AA) residues. GD and ID: Gastric and intestinal digests; GD1 and ID1: Gastric and intestinal digest fractions (>10 kDa), respectively; GD2 and ID2: Gastric and intestinal digest fractions (3–10 kDa), respectively; GD3 and ID3: Gastric and intestinal digest fractions (<3 kDa), respectively.

Peptides from SPC and its digestion fractions were analyzed using the PEAKS Studio V11.5 engine, which performs de novo sequencing by computing the optimal sequences from all possible combinations of AA based on their MS/MS spectra [42]. Results are summarized in Figure 5. After gastric and intestinal digestion, an increase in peptide count was observed, especially in the GD and ID fractions. Additionally, the proportion of medium-length peptides in the GD3, ID2, and ID3 fractions increased compared to the initial SPC sample. In contrast, the fraction of short peptides remained relatively low and constant across samples, while the number of long peptides decreased relatively compared to the initial sample. Following LC-MS/MS analysis, de novo peptides derived from the intestinal digest fraction were compared with those from the SPC to identify SGID-resistant peptides. A total of 416 peptides were identified as resistant, with a cosine similarity value greater than 0.95 and a maximum sequence length difference of 8 AA.

### 3.5. In Silico Analysis

#### 3.5.1. Properties of Resistant Peptides with α-Amylase Inhibitory Potential

A key approach for identifying novel enzyme inhibitors involves analyzing the AA at the enzyme’s active site that interacts with inhibitory peptides. In this study, we classified the identified peptides based on the AA type and their positions at the ultimate (C1 and N1), penultimate (C2 and N2), and antepenultimate (C3 and N3) sites, as documented for α-amylase inhibitory peptides [25]. Of the 416 identified peptides, 17.8% did not meet any criteria, 33.7% met one, 32% met two, 7% met three, and the remaining 9.5% met four or more criteria (Supplementary Materials, Sheets S1 and S2). The physicochemical properties of medium- and long-resistant peptides (selected for meeting ≥3 and ≥4 selection criteria, respectively) were determined using in silico methods, as summarized in Supplementary Materials, Sheet S3.

The MWs of medium and long peptides ranged from 845 to 1211 g/mol and 1178 to 1718 g/mol, respectively. Their pI values varied between 3.43 and 12.10, with 8 peptides classified as physiologically neutral (pI 6.77–7.00). Regarding net charge, 10 peptides were neutral, while 13 exhibited a net positive charge due to the presence of basic AA, such as arginine (R), lysine (K), and histidine (H) [43]. These positively charged peptides are of particular interest, as their side chains may interact with the negatively charged catalytic residues of α-amylase, potentially inhibiting its activity [25]. The remaining peptides carried a negative charge due to acidic AA, such as aspartic acid (D) and glutamic acid (E) [43]. Many selected peptides demonstrated good solubility, a critical feature for oral administration, as it facilitates their incorporation into food formulations and enhances absorption, distribution, and therapeutic efficacy [44,45]. Toxicity assessments revealed that none of the peptides exhibited toxicity in the Ames assay, indicating a low carcinogenic potential. Most peptides had very low toxicity probabilities (0–0.3), except for PCAESLYQSK, which showed a medium probability (0.3–0.7). For rat oral acute (ROA) toxicity, a key safety metric, most peptides exhibited low probabilities, except for RHWLPR and LLFPMSR, which showed medium probabilities. Additionally, none of the peptides displayed allergenic structural features, which is crucial for preventing adverse reactions from dietary proteins [28]. These properties make the peptides promising candidates for incorporation into functional foods with potential antidiabetic effects.

#### 3.5.2. Bioactivity and Bioavailability of Peptides

There is significant interest in resistant peptides due to their potential bioactivity and bioavailability. In this context, PeptideRanker employs a neural network trained with five-fold cross-validation to identify peptides with high bioactivity potential. It predicts a likelihood of bioactivity (ranging from 0 to 1) for each peptide, with values closer to 1 indicating higher confidence in their bioactivity [29]. Of the 416 resistant peptides identified, 13 were classified as highly probable (>0.75) to be bioactive. The top three sequences with the highest scores were QCCDFMK, HGGGGGGFGGGGFSR, and KCCDMMK, as shown in Table 1.

Using the PreAIP tool to predict anti-inflammatory peptides, the sequences QCCDFMK, KCCDMMK, and EWGGGGCGGGGGVSSLR were identified with high probabilities (threshold ≥ 0.47). Seven peptides were classified as medium (0.39 ≤ value < 0.47), two as low (0.34 ≤ value < 0.39), and the sequence HGGGGGGFGGGGFDK was determined not to be anti-inflammatory.

Conversely, peptide bioavailability reflects effective utilization and intact systemic circulation after oral ingestion [46]. To determine drug-likeness, the logarithm of the octanol/water distribution coefficient (log *P*_o/w_) and topological polar surface area (TPSA) were analyzed. Log *P*_o/w_ affects membrane permeability and macromolecular interactions, while TPSA reflects polarity, influencing solubility and membrane crossing [27]. The Pfizer Rule evaluation indicated that the peptides do not meet the criteria of high log *P*_o/w_ (>3) or low TPSA (<75), suggesting they are unlikely to accumulate in biological systems or interact negatively with cellular components, making them suitable for drug development [47]. To evaluate absorption, oral drugs must pass through intestinal membranes via diffusion or transport mechanisms, directly impacting bioavailability. Caco-2 cell lines, which mimic human intestinal cells, are used to assess drug permeability, while human intestinal absorption (HIA) is a key parameter for oral drug efficacy. Caco-2 permeability values below −5.15 log cm/s and HIA probabilities between 0.7 and 1.0 indicate low absorption [27].

In this context, the selected peptides (Table 1) showed low permeability, with mean values of −7.17 log cm/s and 0.92, respectively. To assess distribution, plasma protein binding (PPB) is critical for drug uptake and systemic circulation, influencing pharmacodynamics and oral bioavailability [27]. All evaluated peptides would be distributed adequately via intravenous administration, as they exhibited PPB values below 90%. The selected peptides displayed very low toxicity probabilities (0–0.3) in the rat oral acute toxicity (ROA) assay, except for PSPSLVWR, RHWLPR, and YNLPMLR, which showed medium probabilities (0.3–0.7). Additionally, skin sensitization is a critical concern for topical products [27]. Eight peptides had low probabilities (0–0.3) for skin sensitization, while the remaining five exhibited medium probabilities (0.3–0.7).

Furthermore, preventing potential allergic reactions caused by dietary proteins is essential [28]. None of the peptides exhibited allergenic structural characteristics, making them promising candidates for incorporation into the food and pharmaceutical industries, where they may offer immunomodulatory benefits. Notably, the peptides RHWLPR and SDTLFFAR showed a high probability of being CPP.

### 3.6. Molecular Docking

#### 3.6.1. SGID-Resistant Peptides and α-Amylase

Molecular docking was performed to evaluate the binding capacity of 10 selected peptides (5 medium-sized and 5 long) (Supplementary Materials, Sheet S3) with the enzyme α-amylase. The affinity range of the peptides toward α-amylase was limited, with most medium-sized peptides exhibiting binding energies similar to the standard acarbose (Precose, PubChem CID 444254) (−8.9 kcal/mol), while long peptides showed undetectable affinity, except for QLSNLEQSLSDAEQR. Binding energy, a crucial indicator of ligand–receptor interactions, reflects the stability of the complex, with lower binding energy indicating greater stability [43]. In general, selected peptides showed affinity values similar to those reported in other studies for α-amylase inhibitory peptides [43], ranging from −9.2 kcal/mol and −7.0 kcal/mol. These values suggested that SI peptides had the potential to effectively inhibit α-amylase activity. The peptides were found to bind to both catalytic and substrate-binding residues (Table 2), as reported in the literature [25]. Among all evaluated peptides, RHWLPR showed the strongest interaction with α-amylase, followed by RATVSLPR and QLSNLEQSLSDAEQ.

**Figure 6:**
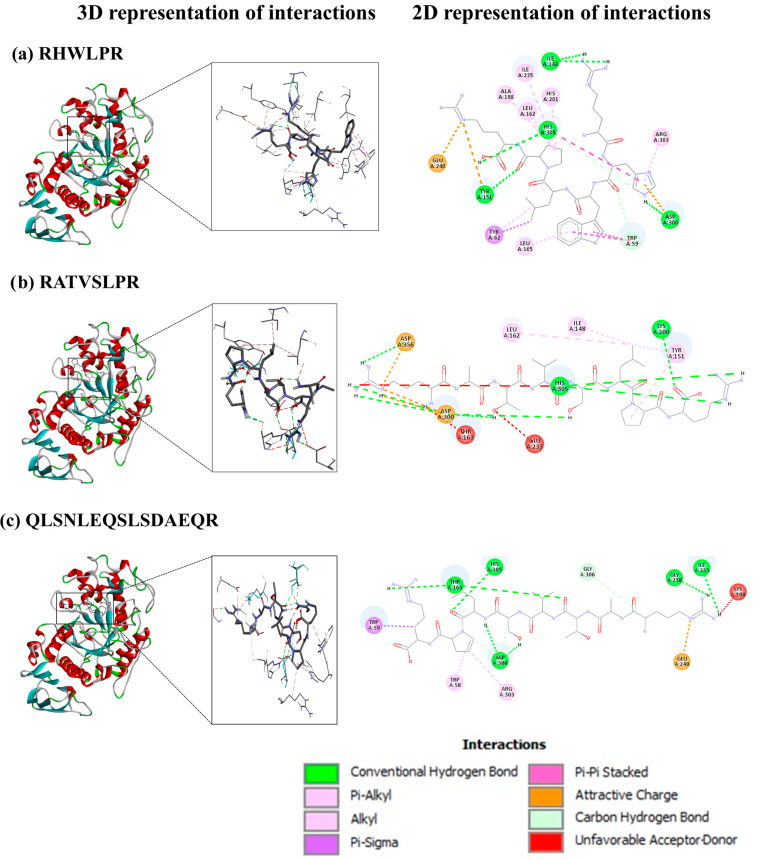
Molecular interactions between ligands and the receptor. (**a**) RHWLPR, (**b**) RATVSLPR, and (**c**) QLSNLEQSLSDAEQR binding to α-amylase. The left part refers to the 3D representation, and the right part refers to the 2D representation of interactions.

The molecular interactions between the catalytic and substrate-binding residues of α-amylase and the peptides with the highest binding affinities were analyzed to elucidate their inhibitory mechanisms. Figure 6 illustrates how RHWLPR, RATVSLPR, and QLSNLEQSLSDAEQR fit within the active site of α-amylase (chain A).

RHWLPR (Figure 6a) interacts with α-amylase through six conventional hydrogen bonds involving residues Trp59, Ile148, Tyr151, His305, and Asp300, where Asp300 is a key catalytic residue [25]. Additionally, RHWLPR bound to non-catalytic residues through hydrophobic and electrostatic interactions, including five mixed π/alkyl interactions with Tyr62, Leu165, His201, and Arg303; three alkyl interactions with Leu162, Ala198, and Ile235; and four π interactions with Trp59 and His305. Electrostatic interactions involved two π-charge interactions with Tyr151 and Asp300, as well as one charge interaction with Glu240.

RATVSLPR (Figure 6b) formed eight conventional hydrogen bonds with Lys200, Asp300, His305, and Asp356, stabilized by hydrophobic interactions, including two mixed π/alkyl interactions with Tyr151 and two alkyl interactions with Ile148 and Leu162. Three electrostatic interactions with Asp300 and Asp356 were also observed, though three unfavorable acceptor/donor clashes with Thr163, Glu233, and His305 were noted.

Finally, QLSNLEQSLSDAEQR (Figure 6c) interacted with α-amylase through eight conventional hydrogen bonds with Thr163, Ile235, Gly238, Asp300, and His305 and one unconventional hydrogen bond with Gly306. Hydrophobic interactions included two mixed π/alkyl interactions with Trp58 and Trp59 and one alkyl interaction with Arg303, alongside an electrostatic interaction with Glu240. However, two unfavorable acceptor/donor clashes with Thr163 and Lys200 were observed, potentially affecting binding affinity.

It is well established that the stability of the ligand–receptor complex is influenced by different molecular interactions, including the number of hydrogen bond interactions, which are directly proportional to the stability and play a critical role in the activation or inhibition of the target receptor, as well as hydrophobic interactions, which also contribute to the stabilization [32]. The lower affinity of RATVSLPR and QLSNLEQSLSDAEQR compared to RHWLPR may result from unfavorable interactions between their functional groups and α-amylase active site residues, introducing steric barriers that reduce binding stability, ligand–receptor binding effectiveness, and complex formation [48]. However, these unfavorable interactions could enhance the peptides’ inhibitory capacity by inducing conformational changes in the 3D structure of α-amylase. Such structural modifications may impair the enzyme’s ability to hydrolyze starch into molecules like maltodextrins, maltose, and glucose.

Structure–activity relationship analyses have identified key chemical features essential for α-amylase inhibition, including aromatic rings, hydrophobic substituents, polar functional groups, stereochemical aspects, and the type and position of AA [25,48]. The peptide RHWLPR, which met multiple criteria related to AA type and position [25], demonstrated the highest affinity for α-amylase. The NH=C(NH_2_)_2_ group of Arg (R) residues at the N1 and C1 positions formed hydrogen bonds with Tyr151 and Ile148, respectively, while the imidazole group of His (H) at the C2 position interacted hydrophobically and stably with Leu162, Ala198, His201, and Ile235. The indole group of Trp (W) at the C3 position, with its electron-dense aromatic system, further stabilized the interaction through a π/alkyl hydrophobic bond and two π hydrophobic bonds with Trp59 and Leu165, respectively.

Research has identified Trp58, Trp59, Tyr62, His299, and Asp300 as the top five critical hotspots for α-amylase interactions with inhibitory peptides [25]. The SGID-resistant peptides interacted with all these residues except His299, indicating their potential as effective α-amylase inhibitors.

#### 3.6.2. SGID-Resistant Peptides and TLR4/MD-2 Complex

TLR4, a key protein in immune cells like macrophages and dendritic cells, recognizes harmful substances, responding to external factors (e.g., bacterial toxins) and internal signals from damaged cells. By forming a complex with MD2, TLR4 enhances its threat detection capability [49]. The TLR4/MD-2 complex specifically identifies LPS or lipid A in Gram-negative bacteria, triggering cytokine production and innate immune responses [50]. Dysregulation of TLR4 signaling is linked to conditions such as sepsis, intestinal inflammation, diabetes-related hypertension, pregnancy complications, drug addiction, and COVID-19, often involving excessive NO and pro-inflammatory cytokines that cause inflammation and tissue damage. Thus, TLR4/MD-2 inhibitors hold significant therapeutic potential for these diseases [49].

**Figure 7:**
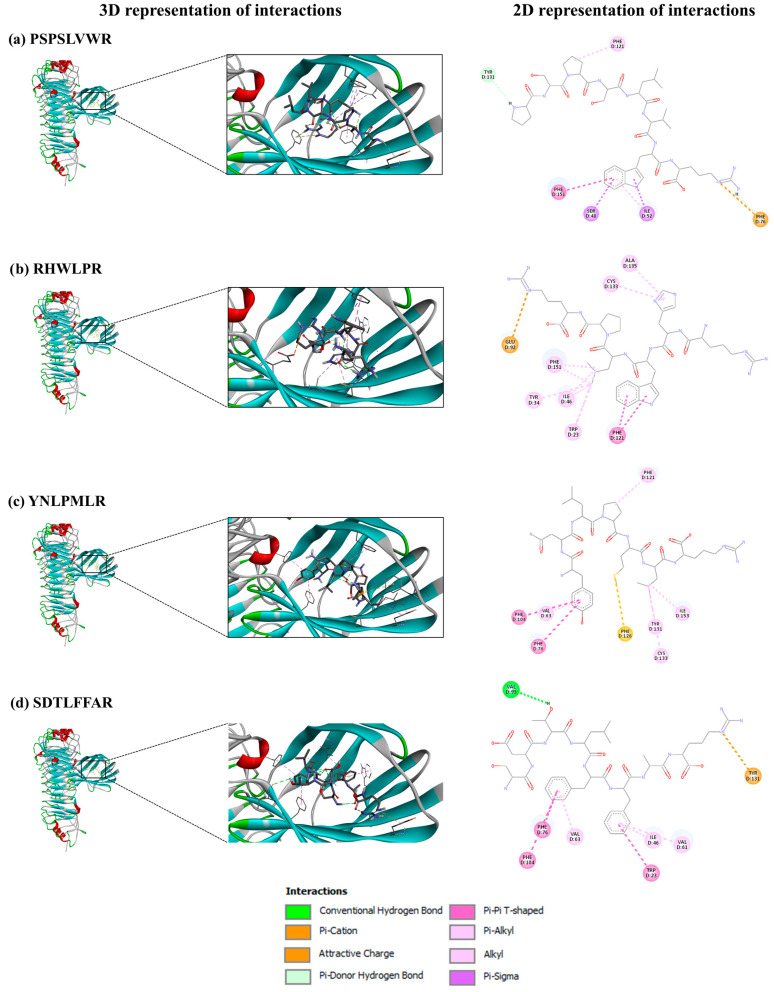
Molecular interactions of (**a**) PSPSLVWR, (**b**) RHWLPR, (**c**) YNLPMLR, and (**d**) SDTLFFAR binding to the TLR4)/MD-2 complex. The left section refers to a 3D representation, and the right section refers to the 2D representation of interactions.

A molecular docking study with 13 selected peptides, as shown in Table 3, revealed affinities for the TLR4/MD-2 complex ranging from −8.5 to −5.5 kcal/mol, slightly lower than immunomodulatory pea peptides (−9.20 to −7.30 kcal/mol) [32]. Structure–activity relationship analyses indicate that effective immunomodulators should include hydrophobic residues (V, G, L, P, F, Y, W, and C) and residues like Q, E, N, or D. Active sequences often have repeated P and E, with Y and K at the N-1 and C-1 positions, and R at both ends [51]. Thus, the sequences PSPSLVWR, RHWLPR, YNLPMLR, and SDTLFFAR were selected to visualize molecular interactions with the TLR4/MD-2 complex, based on their properties: they exhibit hydrophobicity percentages of ≥50%; contain the mentioned amino acids in their structure, as shown in Figure 7; and show medium anti-inflammatory scores in PreAIP (Table 1) with strong receptor binding affinities.

PSPSLVWR (Figure 7a) interacts with the TLR4/MD-2 complex through several key bonds. The –NH_2_ group in the pyrrolidine ring of the P residue at the N-1 position forms a π-donor hydrogen bond with Tyr131, while the P residue at the N-3 position interacts with Phe121 via a mixed π/alkyl hydrophobic bond. The W residue at the C-2 position plays a critical role, as its indole ring connects with Ser48 and Ile52 through three mixed π/alkyl hydrophobic bonds. Additionally, its benzene ring forms an electrostatic π-charge interaction with Phe76 and two interactions with Phe151: one non-classical hydrogen bond and one π-hydrophobic bond.

PSPSLVWR demonstrates significant anti-inflammatory potential. First, its binding to Phe121 of the TLR4/MD-2 complex mirrors the mechanism of the PIP2 peptide, which inhibits LPS-induced production of pro-inflammatory factors by preventing TLR4/MD-2 complex formation through hydrophobic interactions with Phe121 of MD2 [52]. Second, its interaction with Phe151 is crucial for inhibiting the TLR4/MD-2 complex, as evidenced by studies with the FP13-17 monosaccharide-based lipid A analogue [53] and the inactive opioid isomer (+)-naltrexone [54].

RHWLPR (Figure 7b) binds to the TLR4/MD-2 complex through the guanidinium group of R (C-1), forming an electrostatic charge interaction with Glu92. The isobutyl group of L interacts with Phe151, Tyr34, and Trp23 through mixed π/alkyl hydrophobic bonds and with Ile46 via alkyl hydrophobic bonds. The indole ring of W forms two π-hydrophobic bonds with Phe151, while the imidazole ring of H binds to Cys133 and Ala135 through mixed π/alkyl hydrophobic bonds. This interaction may disrupt TLR4/MD-2 dimerization, as the guanidine group of calixarenes similarly binds Glu92, inhibiting dimerization in human and mouse cells [55].

YNLPMLR (Figure 7c) binds through the phenolic group of Y (N-1), forming π-hydrophobic bonds with Phe104 and Phe76 and a mixed π/alkyl hydrophobic bond with Val63. The P residue interacts with Phe121 via a mixed π/alkyl hydrophobic bond, while M forms a sulfur bond with Phe126. The isobutyl group of L binds Tyr131, Cys133, and Ile153 through mixed π/alkyl and alkyl hydrophobic bonds.

SDTLFFAR (Figure 7d) interacts with the TLR4/MD-2 complex through multiple key interactions. The carboxyl group of D forms a hydrogen bond with Val93. The benzene ring of F (C-2) forms π-hydrophobic bonds with Phe76 and Phe104 and a mixed π/alkyl hydrophobic bond with Val73. The benzene ring of F (C-3) interacts with Trp23 via a π-hydrophobic bond and with Ile46 and Val61 through mixed π/alkyl hydrophobic bonds. The guanidinium group of R forms an electrostatic π-charge interaction with Tyr131.

The last two peptides also exhibit anti-inflammatory potential by binding to the Phe76 residue of the TLR4/MD-2 complex, similar to the interaction of the benzene ring of naltrexone [54]. Additionally, YNLPMLR binds to the Phe121 residue and the Phe126 loop of MD-2, both critical for TLR4/MD-2 dimerization [50] and LPS binding to the TLR4/MD-2 complex [49], respectively. Similarly, SDTLFFAR binds to the Ile46 residue, mirroring the interaction of the FP13-17 lipid A analogue [53].

## 4. Conclusions

This study demonstrates that recovery proteins from SIPC, when hydrolyzed through SGID, yield peptide fractions with dual bioactive properties: antidiabetic and immunomodulatory. In vitro studies revealed that peptide fractions from both gastric and intestinal digests inhibit α-amylase activity, with the intestinal digest < 3 kDa fraction showing the highest inhibition (45% at 20 mg/mL). Additionally, both gastric and intestinal < 3 kDa fractions significantly reduced NO production in LPS-challenged RAW 264.7 macrophages. These findings suggest that consuming SI protein concentrates could be beneficial for functional foods aimed at preventing starch breakdown, reducing blood glucose release, and mitigating oxidative tissue damage and inflammation in conditions requiring immune modulation.

In silico analyses identified SGID-resistant peptides, such as RHWLPR, RATVSLPR, and QLSNLEQSLSDAEQR, which exhibit strong α-amylase inhibitory activity. These peptides interact with catalytic residues (Glu233 and Asp300) and critical hotspots (Trp58, Trp59, and Tyr62) through hydrogen bonds, hydrophobic interactions, and electrostatic forces. Also, in silico studies of SGID-resistant peptides PSPSLVWR, RHWLPR, YNLPMLR, and SDTLFFAR confirmed their bioactivity and bioavailability, demonstrating low toxicity, no allergenic properties, good distribution characteristics, and suitability for drug development. Notably, RHWLPR and SDTLFFAR showed a high probability of being cell-penetrating peptides, enhancing their therapeutic potential. However, it is important to acknowledge that in silico tools have limitations, as they provide theoretical predictions that require experimental validation, using, for example, a cellular monolayer model to determine whether brush border enzymes can alter the activity and bioavailability of the identified peptides.

This study lays the foundation for developing innovative, peptide-based strategies to address diabetes and inflammation through dietary interventions. Future research should focus, on the one hand, on molecular dynamics simulations to confirm the stability of peptide-enzyme molecular interactions. In addition, the chemical synthesis of identified SGID-resistant peptides, particularly RHWLPR, should be carried out to validate their bioactivity, bioavailability, and mechanisms of action. Additionally, their incorporation into functional foods or nutraceuticals should be explored to assess efficacy and safety in food applications. These efforts will advance the development of peptide-based therapies and functional ingredients for managing metabolic and inflammatory conditions.

## Figures and Tables

**Table 1:** Bioactivity and bioavailability of sacha inchi peptides using in silico tools.

CPP ^e^		Aller. ^d^	Toxicity ^c^		Distribution ^c^	Absorption ^c^		Drug-likeness ^c^	PreAIP ^b^	Ranks ^a^	Peptide
Prob.	Class.		Skin	ROA	PPB	HIA	Caco-2	Pfizer Rule			
0.16	Non-CPP	No ev.	0.21	0.04	0.16	0.92	−7.10	Accepted	0.61	0.92	QCCDFMK
0.40	Non-CPP	No ev.	0.48	0.19	0.31	1.00	−6.90	Accepted	0.38	0.87	HGGGGGGFGGGGFSR
0.42	Non-CPP	No ev.	0.25	0.01	0.17	0.97	−6.77	Accepted	0.66	0.86	KCCDMMK
0.22	Non-CPP	No ev.	0.12	0.45	0.23	0.99	−6.97	Accepted	0.42	0.85	PSPSLVWR
0.12	Non-CPP	No ev.	0.59	0.01	0.20	0.97	−7.52	Accepted	0.56	0.85	EWGGGGCGGGGGVSSLR
0.33	Non-CPP	No ev.	0.32	0.14	0.23	0.83	−7.52	Accepted	0.42	0.84	SGGFGGNFGNR
0.72	CPP	No ev.	0.15	0.63	0.16	0.80	−6.24	Accepted	0.38	0.83	RHWLPR
0.07	Non-CPP	No ev.	0.24	0.08	0.16	0.92	−7.60	Accepted	0.45	0.81	SDWPELLGR
0.42	Non-CPP	No ev.	0.63	0.00	0.12	0.90	−7.84	Accepted	0.40	0.79	SGGGGGGLGSGGSLR
0.02	Non-CPP	No ev.	0.14	0.36	0.15	0.86	−6.87	Accepted	0.42	0.79	YNLPMLR
0.39	Non-CPP	No ev.	0.5	0.07	0.31	1.00	−7.24	Accepted	0.32	0.79	HGGGGGGFGGGGFDK
0.70	CPP	No ev.	0.09	0.13	0.19	0.89	−7.63	Accepted	0.42	0.76	SDTLFFAR
0.11	Non-CPP	No ev.	0.19	0.47	0.20	0.94	−7.00	Accepted	0.43	0.75	LLFPMSR

^a^ PeptideRanker (http://distilldeep.ucd.ie/PeptideRanker/, accessed August 2024) ranks peptides by the predicted probability that the peptide will be bioactive. ^b^ PreAIP was used to estimate the probability of the peptides to exert anti-inflammatory effects (http://kurata14.bio.kyutech.ac.jp/PreAIP/, accessed August 2024). ^c^ Bioavailability was evaluated using ADMETlab 2.0 (https://admetmesh.scbdd.com/service/screening/index, accessed August 2024), which includes drug-likeness (Pfizer Rule), absorption (Caco-2 permeability and human intestinal absorption [HIA] scores), distribution (plasma protein binding [PPB] score), and toxicity (rat oral acute [ROA] and skin sensitization scores). ^d^ Allergenicity (Aller.) was evaluated using AllerCatPro 2.0 (https://allercatpro.bii.a-star.edu.sg/). ^e^ MLCPP 2.0 was used to estimate the classification (Class.) and probability (Prob.) of cell-penetrating peptides (CPP) (https://balalab-skku.org/mlcpp2/, accessed August 2024). Conventions: A (alanine); C (cysteine); D (aspartic acid); E (glutamic acid); F (phenylalanine); G (glycine); H (histidine); K (lysine); L (leucine); M (methionine); N (asparagine); P (proline); Q (glutamine); R (arginine); S (serine); T (threonine); V (valine); W (tryptophan); and Y (tyrosine). Tools were accessed in August 2024.

**Table 2:** Binding affinity of ligand–receptor interactions (kcal/mol) and interactions of sacha inchi peptides with α-amylase hotspots.

Bound Sites	Affinity	Peptide Sequence
Trp58, Trp59 **, Thr163, Asp197 *, Lys200, His201, Ile235, Asp300 *, Ala307, Asp356	−7.4	LHALEDPNR
Trp59 **, Tyr62 **, Tyr151 **, Leu162 Leu165, Ile168, Ala198, His201, Ile235, Glu240, Asp300 *, Arg303, His305	−9.2	RHWLPR
Thr163, Leu165, Ile 235, Leu237, Glu240, Lys257, Asp300 *, Asp356	−7.0	LPTQSWKVPR
Ile148, Tyr151**, Leu162, Thr163, Lys200, Glu233 *, Asp300 *, His305, Asp356	−8.1	RATVSLPR
Trp59 **, His101, Tyr151 **, Leu162, Thr163, Lys200, His201, Ile235, Glu240, His299, Asp300 *, Hys305, Gly306	−7.6	LSASGHVVLR
-	n.d	QNSNLQKSLSDAEQR
Trp58, Trp59, Thr163, Lys200, Ile235, Gly238, Glu240, Asp300 *, Arg303, His305, Gly306	−7.8	QLSNLEQSLSDAEQR
-	n.d	LQSNLQKSLSDAEQR
-	n.d	SPSNLQKSLSDAEQR
-	n.d	KNSNLQQSLSDAEQR

* Catalytic residues. ** Substrate-binding residues. n.d. Not detected by molecular docking. Conventions: A: alanine; C: cysteine; D: aspartic acid; E: glutamic acid; F: phenylalanine; G: glycine; H: histidine; K: lysine; L: leucine; M: methionine; N: asparagine; P: proline; Q: glutamine; R: arginine; S: serine; T: threonine; V: valine; W: tryptophan; Y: tyrosine.

**Table 3:** Binding affinity of ligand–receptor interactions (kcal/mol) between sacha inchi peptides and the TLR4/MD-2 complex.

Neutral ^e^ (%)	Basic ^d^ (%)	Acidic ^c^ (%)	Hydrophobic ^b^ (%)	MW ^a^	Length ^a^	Affinity	Peptide
42.86	14.29	14.29	28.57	874.07	7	−5.50	QCCDFMK
73.33	13.33	0.00	13.33	1263.28	15	−8.50	HGGGGGGFGGGGFSR
28.57	28.57	14.29	28.57	858.14	7	−4.80	KCCDMMK
25.00	12.50	0.00	62.50	941.08	8	−7.60	PSPSLVWR
70.59	5.88	5.88	17.65	1492.58	17	−6.80	EWGGGGCGGGGGVSSLR
72.73	9.09	0.00	18.18	1069.09	11	−6.90	SGGFGGNFGNR
0.00	50.00	0.00	50.00	864.01	6	−7.80	RHWLPR
22.22	11.11	22.22	44.44	1072.17	9	−7.30	SDWPELLGR
81.25	6.25	0.00	12.50	1175.21	15	−6.50	SGGGGGGLGSGGSLR
28.57	14.29	0.00	57.14	906.11	7	−7.80	YNLPMLR
66.67	13.33	6.67	13.33	1263.28	15	−6.40	HGGGGGGFGGGGFDK
25.00	12.50	12.50	50.00	956.05	8	−7.80	SDTLFFAR
14.29	14.29	0.00	71.43	863.08	7	−7.60	LLFPMSR

^a^ Length and molecular weight (MW, g/mol) were estimated using PepCalc (http://pepcalc.com/, accessed August 2024). ^b^ Hydrophobic amino acids include A (alanine), C (cysteine), F (phenylalanine), G (glycine), H (histidine), L (leucine), M (methionine), P (proline), V (valine), W (tryptophan), and Y (tyrosine). ^c^ Acidic amino acids include D (aspartic acid) and E (glutamic acid). ^d^ Basic amino acids include K (lysine) and R (arginine). ^e^ Neutral amino acids include N (asparagine), Q (glutamine), S (serine), and T (threonine). Tool was accessed in August 2024.

## Data Availability

The original contributions presented in the study are included in the article/Supplementary Materials; further inquiries can be directed to the corresponding authors.

## Supplementary Materials

The following supporting information can be downloaded at www.mdpi.com/article/10.3390/foods14071231/s1, a Microsoft Excel workbook containing three sheets: Sheet S1: List of medium-sized peptides (5–10 AA) classified by AA type and positions at ultimate (C1: L, W, S; N1: P, A, L, M, H, R), penultimate (C2: P, L, M, N; N2: L, I, P, V, C, H), and antepenultimate (C3: R, P, M, G, K; N3: P, I, A, W, H) locations. Sheet S2: List of long-sized peptides (≥10 AA) classified by AA type and positions at ultimate (C1: L, W, S; N1: R, S, Q), penultimate (C2: R, S, Q, Y; N2: D, M, V, N, Q, S), and antepenultimate (C3: Q, E; N3: A, G, S) locations. Sheet S3: In silico properties of potential α-amylase inhibitory peptides from sacha inchi meal.

## Abbreviations

The following abbreviations are used in this manuscript:

AA	Amino acids
ALC	Average local confidence
ANOVA	Analysis of variance
DM2	Diabetes mellitus type 2 diabetes
DMEM	High-glucose Dulbecco’s modified Eagle’s medium
DPPH	2,2-diphenyl-1-picrylhydrazyl
DPP-IV	Dipeptidyl peptidase-IV
ELISA	Enzyme-linked immunosorbent assay
FAO	Food and Agriculture Organization
FBS	Fetal bovine serum
HPLC-MS/MS	High-performance liquid chromatography coupled with Tandem mass spectrometry
IC50	Half-maximal inhibitory concentration
IL-6	Interleukin-6
LC-MS/MS	Liquid chromatography-Tandem mass spectrometry
LPS	Lipopolysaccharide
MW	Molecular weight
NO	Nitric oxide
PBS	Phosphate-buffered saline
pI	Isoelectric point
RCSB	Research Collaboratory for Structural Bioinformatics
SGID	Simulated gastrointestinal digestion
TLR4/MD-2	Toll-like receptor 4/Myeloid differentiation protein-2
TNF-α	Tumor necrosis factor-α
UN	United Nations
WHO	World Health Organization
